# Supplementary material for: Structural basis of topoisomerase targeting by delafloxacin
Source: Nat Commun. 2025 Jul 1;16:5829. doi: 10.1038/s41467-025-60688-3 (PMC12217528; doi:10.1038/s41467-025-60688-3)

Supplementary Information for

Structural basis of topoisomerase targeting by delafloxacin

Shabir Najmudin[1#], Xiao-Su Pan[1#], Beijia Wang[2#], Lata Govada[2], Naomi E. Chayen[2], Noelia Rubio[3], Milo S.P. Shaffer[3], Henry S. Rzepa[4], L. Mark Fisher[1*] and Mark R. Sanderson[1,2*]

[1]Molecular and Cellular Sciences Section, Neuroscience and Cell Biology Research Institute, City St George's, University of London, Cranmer Terrace, London SW17 0RE, UK.
[2]Division of Systems Medicine, Department of Metabolism, Digestion and Reproduction, Faculty of Medicine, Imperial College London, London W12 0NN, UK.
[3]Departments of Chemistry & Materials Science, Imperial College London, London SW7 2AZ, UK.
[4]Department of Chemistry, Imperial College London, London W12 0BZ, UK.

#Contributed equally.

This file contains:

Supplementary Figures 1 to 10.

Supplementary Tables 1 to 7.

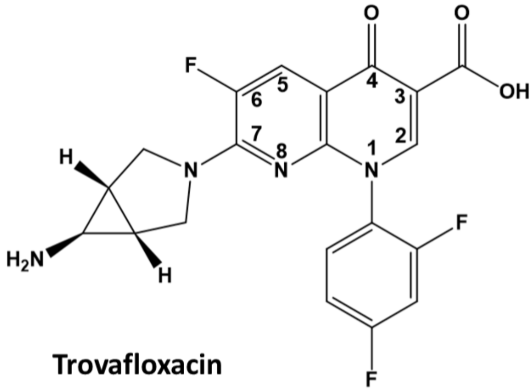

Trovafloxacin

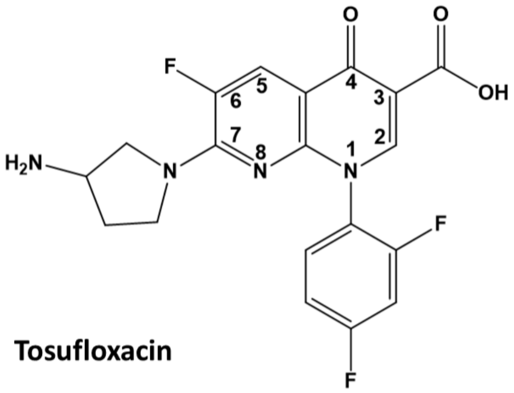

Tosufloxacin

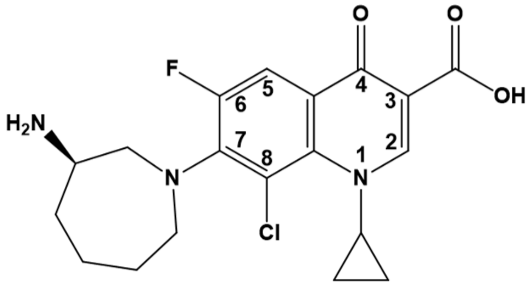

Besifloxacin

Supplementary Fig. 1 Other fluoroquinolones that share with delafloxacin an aromatic ring at N-1 or a chlorine substituent at C-8.

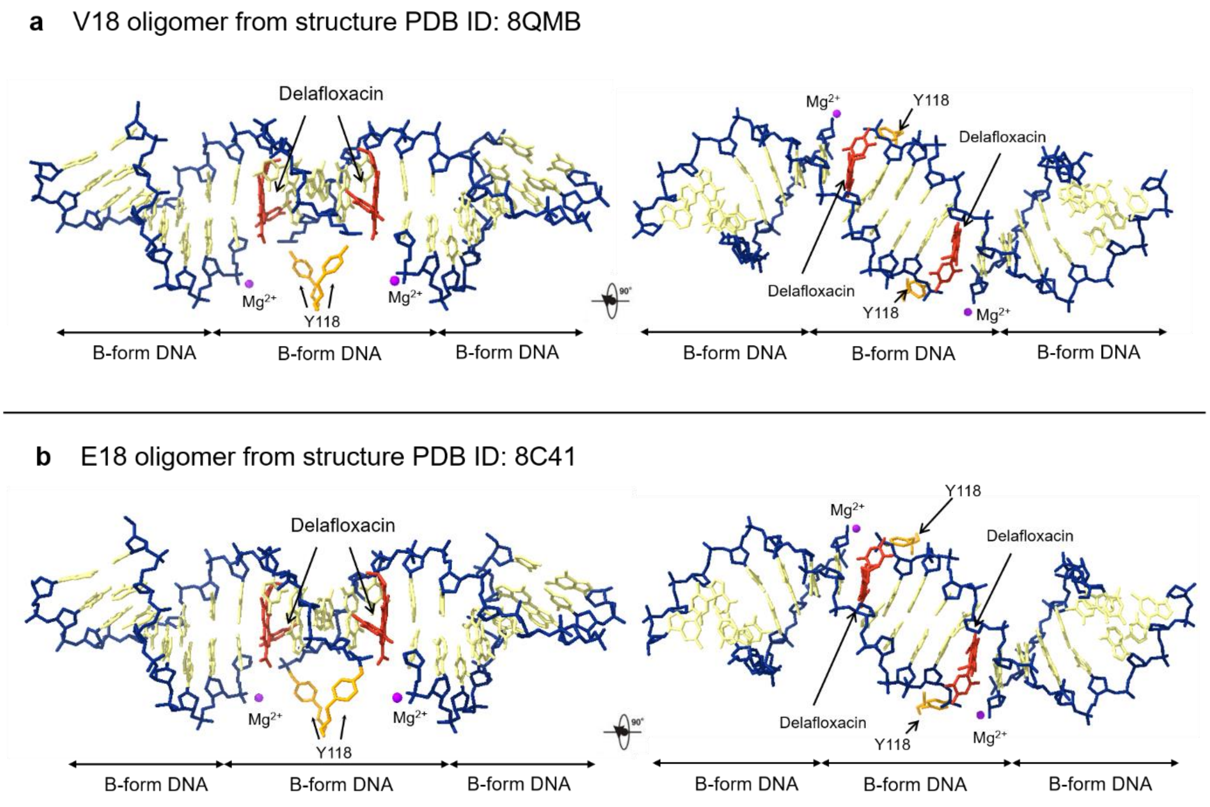

Supplementary Fig. 2 and accompanying video (Supplementary_data_1). The (a)V18 (PDB ID: 8QMB) and (b) E18 (PDB ID: 8C41) G-segment duplex DNAs adopt similar conformations in the topo IV complex stabilized by delafloxacin (in red). Magnesium ions are shown in purple. Both DNA strands are cleaved with the catalytic Y118 ParC residues (shown in orange) covalently linked one to each 5'-end of the 4-bp staggered breaks on complementary strands. DNA analysis with w3DNA software indicates that the V and E sites duplexes are bound in the cleavage complex as B-form DNA.

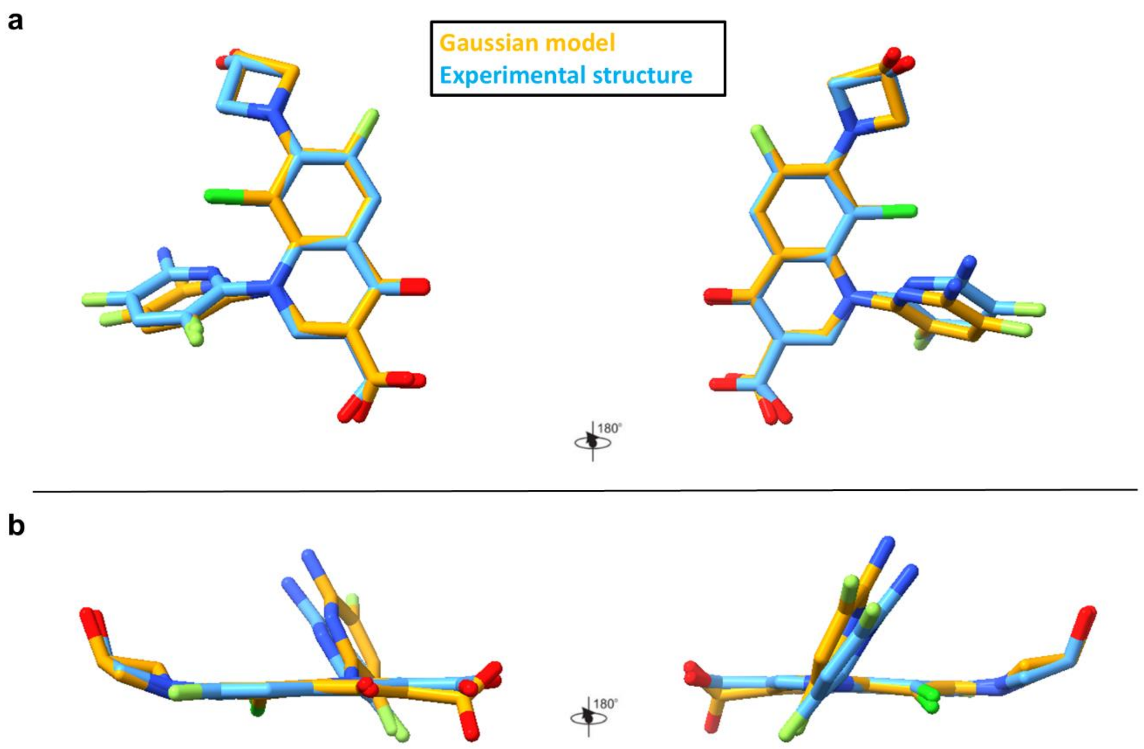

Supplementary Fig. 3 Close similarity of the topo IV-bound delafloxacin (from PDB ID: 8QMB) conformation to that calculated for the free drug using Gaussian 16 (see Methods section and Supplementary Table 2). (a) Views perpendicular to the quinolone plane and (b) views parallel to the quinolone plane.

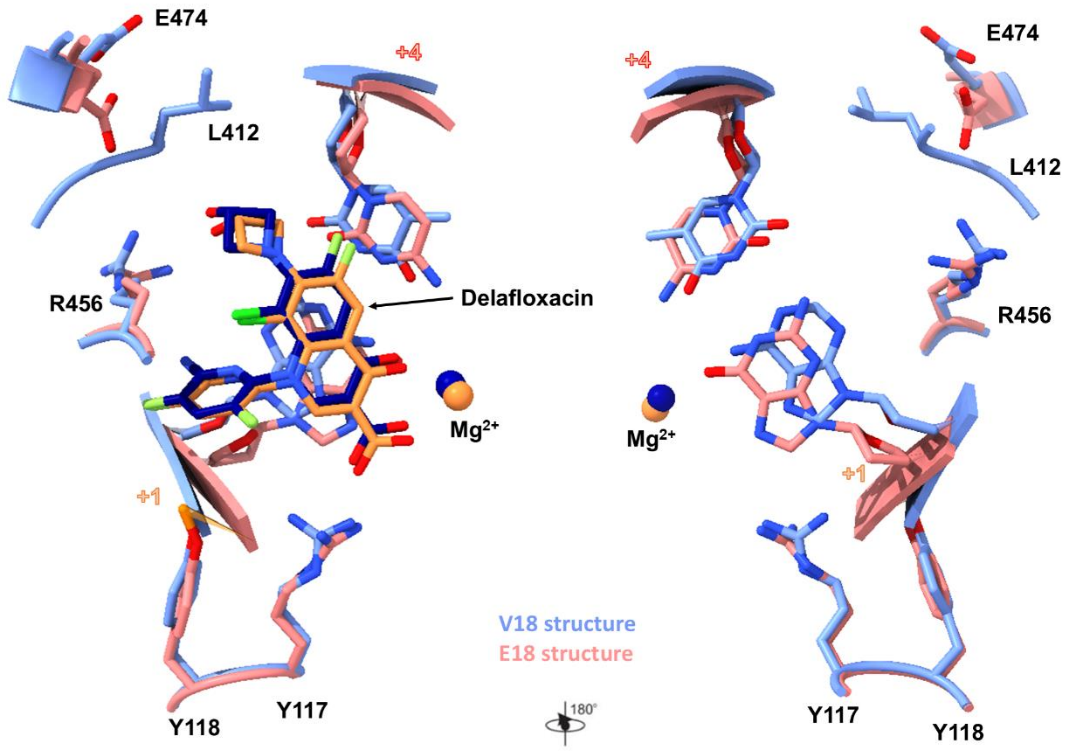

Supplementary Fig. 4 Comparison of delafloxacin binding sites in the cleavage complex with V18 DNA (protein residues and DNA in light blue; delafloxacin and coordinated magnesium ion in dark blue) and with E18 DNA (protein residues and DNA in pink, delafloxacin and coordinated magnesium ion in orange).

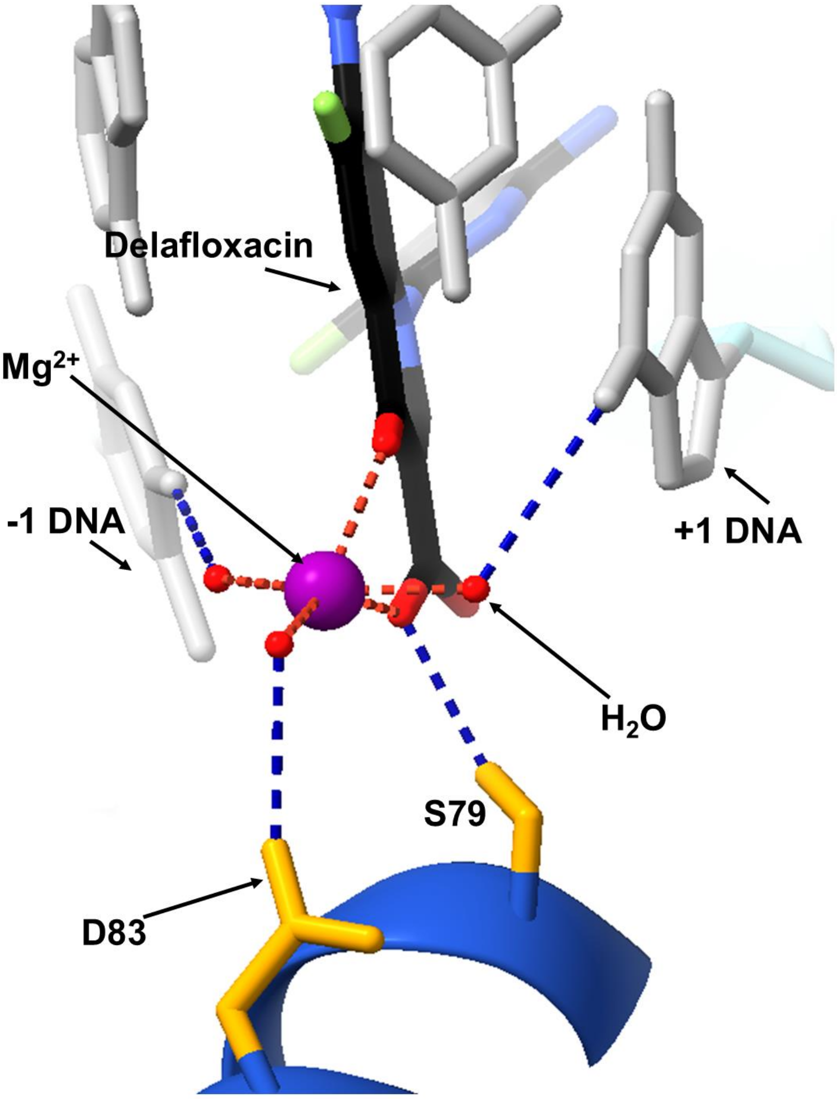

Supplementary Fig. 5 Close-up of the delafloxacin-E18 DNA-cleavage complex (PDB ID: 8C41) shows the DNA-intercalated drug forming three separate water-metal ion links to the -1 and +1 DNA bases and to the side chain of ParC Asp83, plus direct drug binding to ParC Ser79.

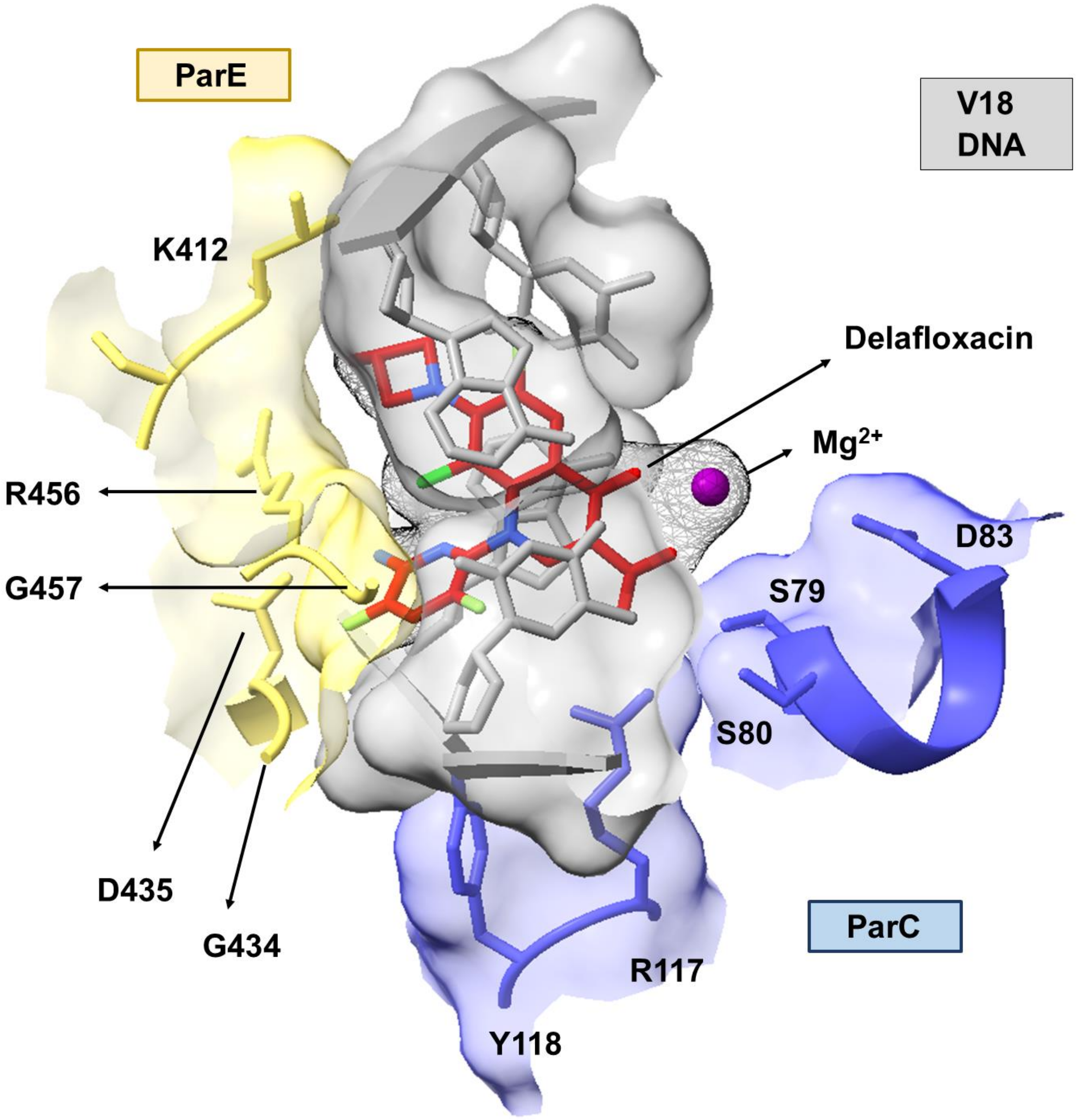

Supplementary Fig. 6 and accompanying video (Supplementary_data_2). The N-1 heteroaromatic ring of delafloxacin is bound in a sub-pocket formed between DNA and ParE (PDB ID: 8QMB). Protein residues and DNA are shown in surface representation, ParC in blue, ParE in yellow and DNA in grey. (See attached movie of the binding pocket).

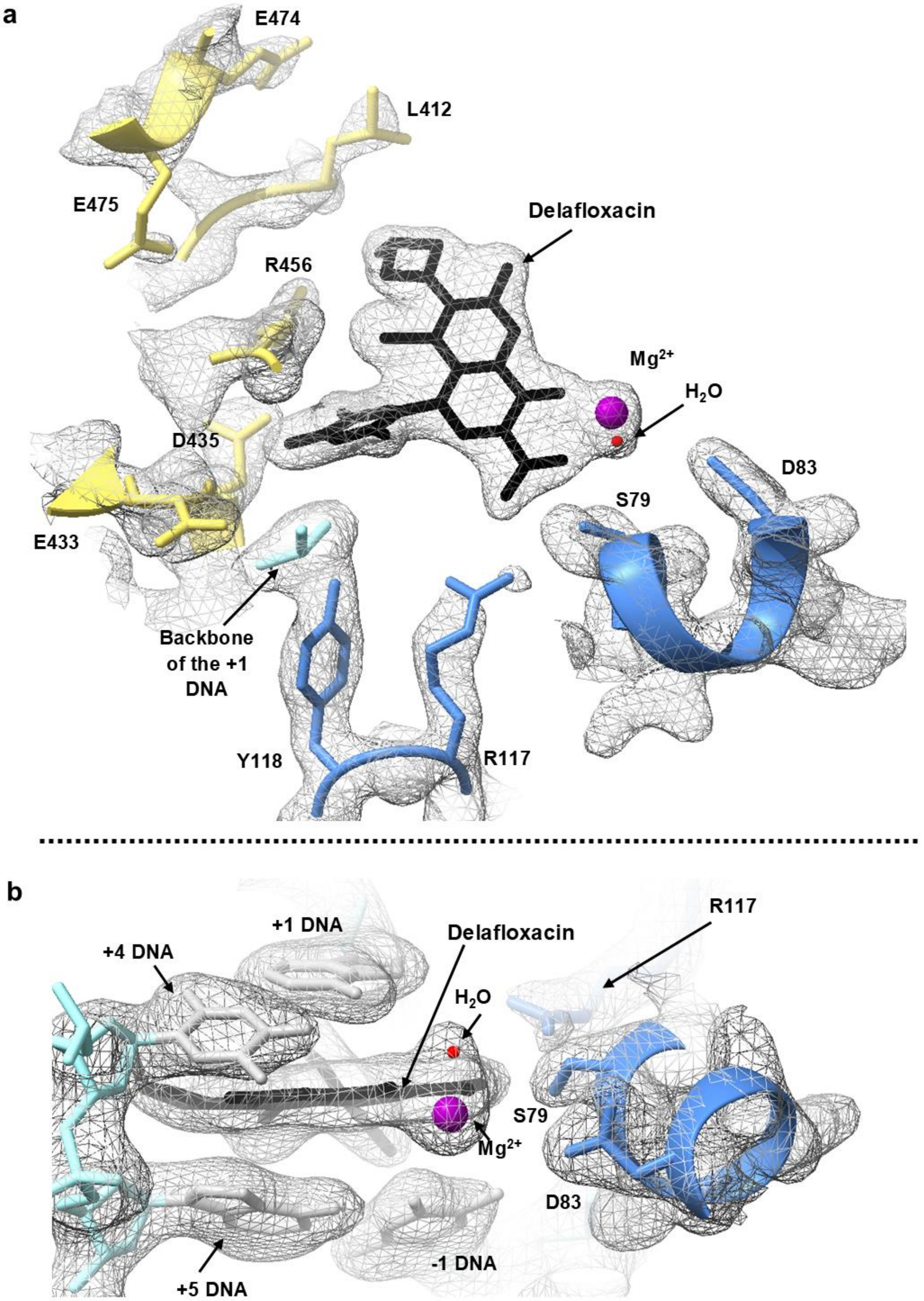

Supplementary Fig. 7 a and **b.** Representative electron density maps for the drug binding site in the topo IV-delafloxacin-DNA cleavage complex (PDB ID: 8QMB). The ParC residues are in blue, the ParE residues in yellow, DNA backbone is in light blue, delafloxacin is in black, magnesium ion is in purple, DNA bases are in silver. (2mFo-DFc map contoured at 1.5σ)

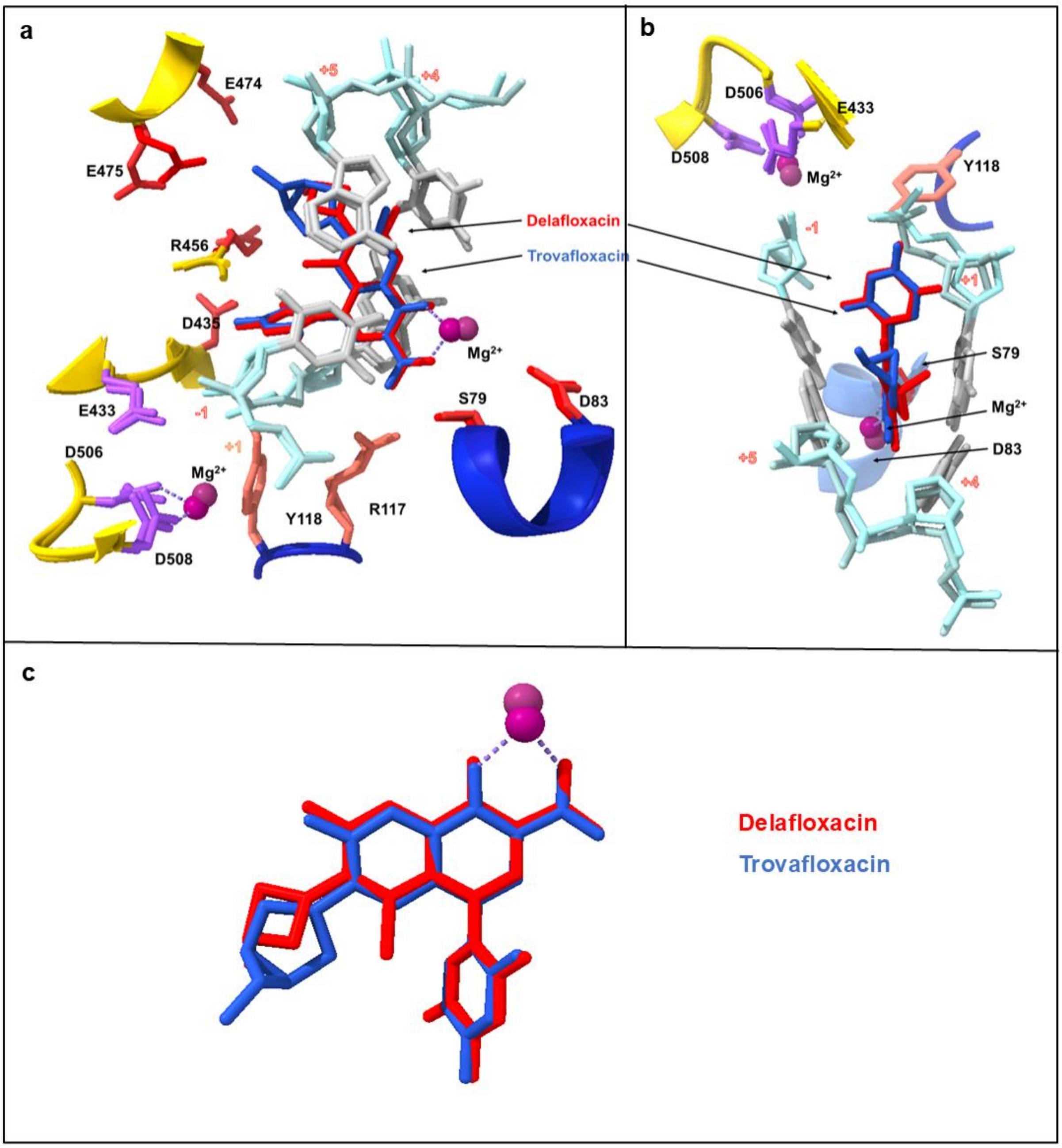

Supplementary Fig. 8 Comparison of E-18- topo IV cleavage complexes formed with delafloxacin and trovafloxacin. **a, b** Orthogonal views of the least-atom least-squares superposition of the active site of the delafloxacin complex (PDB ID: 8C41) with that of the trovafloxacin complex (4KOE) determined at 3.02 Å. **c** Superposition of bound delafloxacin (in red) and bound trovafloxacin (in blue). Magnesium ions are shown in purple.

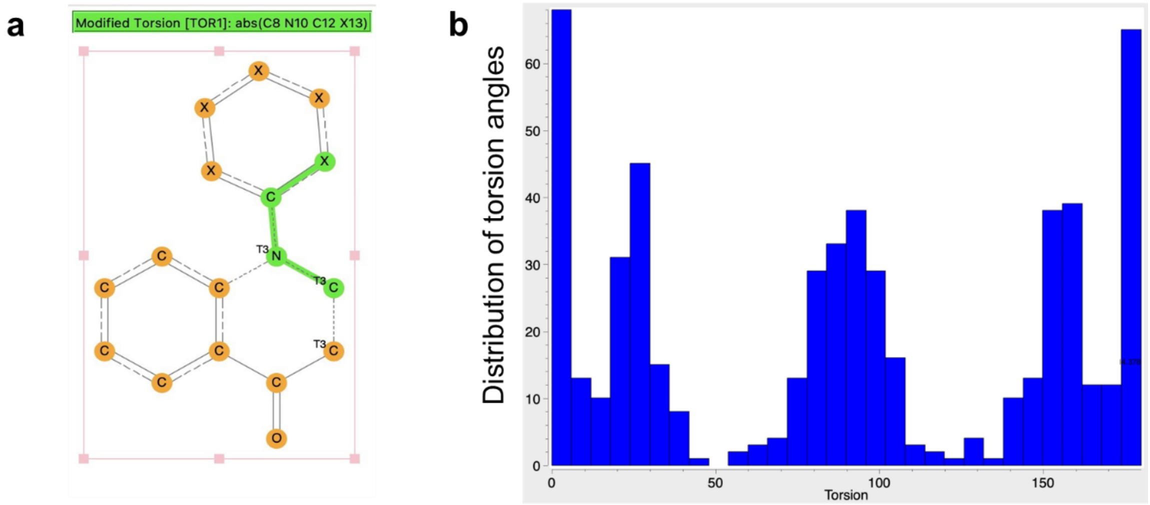

N-1-aromatic quinolone search framework

C8-N10-C12-X13 torsion angle (degrees)

Supplementary Fig. 9 A tilted N-1 aromatic ring is seen in other aromatic systems. A search of the Cambridge small molecule database using the N-1-aromatic-substituted quinolone framework shown in **a** recovered 178 entries that give the distribution of torsion angles shown in **b**. Some molecules are planar (torsion angle at or around 0 or 180 degrees) but for other entries the torsion angles cluster at 20-30-, 80-110- and 150-160-degrees indicating deviation from sp^2 geometry and significant tilting of the N-1 aromatic ring relative to the bicyclic aromatic quinolone nucleus.

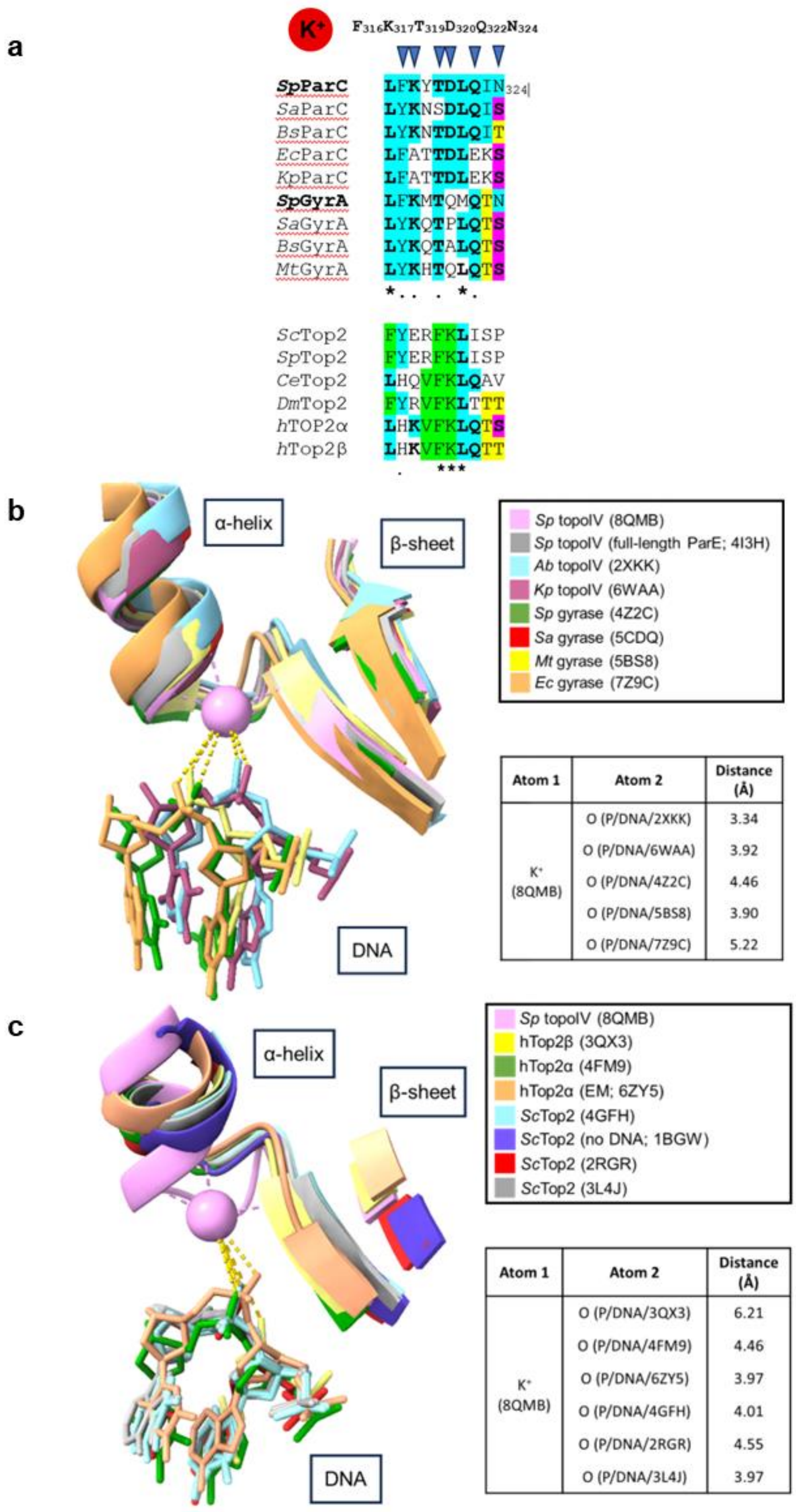

Supplementary Fig. 10 The 'tower' domain potassium binding site: comparison of sequence and structure between prokaryotic and eukaryotic type II topoisomerases. **a**. The *S. pneumoniae* (*Sp*) topo IV K^+-site sequence is highly conserved among bacterial topo IV and gyrase enzymes but differs in eukaryotic type II topoisomerases. *S. pneumoniae* topo IV residues that bind K^+ are indicated by arrows. Sequence

identity with *Sp* ParC is indicated in blue; other sequence identities (not involving pneumococcal ParC) are highlighted in maroon, and yellow. Sequence preferences particular to the eukaryotic enzymes are shown in green. *Staphylococcus aureus* (*Sa*), *Bacillus subtilis* (*Bs*), *Escherichia coli* (*Ec*), *Klebsiella pneumoniae* (*Kp*), *Mycobacterium tuberculosis* (*Mt*), *Saccharomyces cerevisiae* (*Sc*), *Schizosaccharomyces pombe* (*Sp*), *Caenorhabditis elegans* (*Ce*), *Drosophila melanogaster* (*Dm*), and the human topo IIα (hTop2α) and topo IIβ (hTop2β) isoforms. **b**. A helix-loop-β-sheet motif that forms the 'tower' binding site for potassium (pink sphere) in *S. pneumoniae* topo IV is highly conserved in the structures of DNA cleavage complexes of other bacterial topo IV and gyrases. Overlay of these structures (e.g. 4I3H) indicates that the relevant phosphate oxygens of the DNA (between nt 20-21 which are absent in our truncated V18 DNA structure 8QMB) are in range to bind the K^+ ion (dotted lines and distances shown). **c**. Human and yeast Top2 tower domains have a conserved but distinct helix-loop-β-sheet motif with a truncated helix and lacking the pronounced K^+ binding loop of bacterial topo IV and gyrase. Species designations/distances/PDB entries as in **a**, **b** and here include *Acinetobacter baumannii* (*Ab*) topo IV.

Supplementary Table 1 X-ray data collection and refinement statistics.

	Core complex with V18 and delafloxacin, grown in the presence of nucleant (8QMB)	Core complex with V18 and delafloxacin, grown in the absence of nucleant (8QMC)	Core complex with E18 and delafloxacin (8C41)
**Data Collection**			
Space group	$P3_1$ 2 1	$P3_1$ 2 1	$P3_1$ 2 1
Cell dimensions			
a, b, c (Å)	157.22, 157.22, 212.95	157.14, 157.14, 211.64	157.47, 157.47, 212.34
α, β, γ (°)	90.00, 90.00, 120.00	90.00, 90.00, 120.00	90.00, 90.00, 120.00
Resolution (Å)	83.699 – 1.997 (1.997 – 1.997)	64.685 - 2.406 (2.406 – 2.406)	114.746 - 2.394 (2.689 - 2.394)
R_{merge}	0.239 (2.948)	0.341 (2.050)	0.564 (4.363)
Rpim	0.024 (0.326)	0.078 (0.459)	0.073 (0.620)
No. of unique reflections	115609 (6766)	60189 (5142)	70836 (3517)
CC 1/2	1.000 (0.827)	0.997 (0.745)	1.000 (0.830)
Mean(I)/sd(I)	19.0 (2.5)	10.3 (2.0)	8.4 (1.8)
Completeness (%) (Ellipsoidal)	96.6 (82.0)	96.2 (86.2)	96.3 (84.0)
Completeness (%) (Spherical)	56.6 (9.5)	51.5 (10.5)	57.0 (10.0)
Multiplicity	100.1 (82.3)	20.1 (20.8)	60.8 (49.5)
**Refinement**			
Resolution (Å)	83.699 – 2.00	64.685 - 2.41	114.74 – 2.39
No. of reflections all/free	115608/5806	60017/3063	70682/3458
R_{work}/R_{free}	0.169/0.206	0.200/0.251	0.190/0.241
No. of atoms			
Protein	23020	22872	22504
DNA	1139	1139	1138
Ligand/ions	213	175	151
Water	1079	670	418
B factors			
Protein	36	27	55
DNA	32	30	61
Ligand/ions	44	35	55
Water	41	11	28
R.m.s. deviations			
Bond lengths (Å)	0.0124	0.0083	0.0063
Bond angles (°)	2.260	1.530	1.429

Supplementary Table 2 Torsion angles for the off-plane deviations of the N-1 heteroaromatic ring and Cl substituent in delafloxacin.

Torsion angles	Experimental structure	Gaussian 16 model
**Phenyl ring to fluoroquinolone plane (C1-N1-C2-N2)** 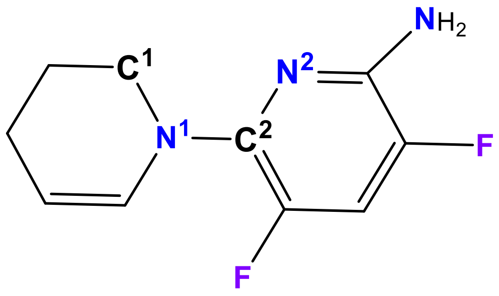	51.2°	50.7°
**Cl to fluoroquinolone plane (C6-C7-C8-Cl)** 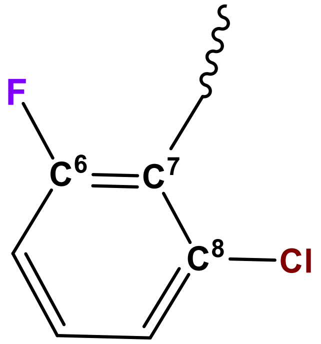	-6.8°	-17.1°

Supplementary Table 3 Structures of bacterial and eukaryotic type II topoisomerases are highly conserved. The Table compares the degree of structural overlap when X-ray or cryoEM structures of various topoisomerases are superimposed on the reference structure of the core DNA complex of *S. pneumoniae* topo IV (9GEF). Overlap is measured by the root mean squares deviation (RMSD) between the two structures. Perfect superposition corresponds to an RMSD value of zero. A value of <2 suggests significant structural conservation; values around 1 indicate substantial structural identity, as visualized locally for the ParC K+ binding site (see Supplementary Fig 7).

Enzyme-DNA complex	Drug	Core	Structure X-ray/EM	PDB code	Alignment RMSD[a] vs 9GEF
*Sp*Topo IV	Yes	Yes $(E_{30}\text{-}C_{55})_2$[b]	X-ray	9GEF	-
*Sp*Topo IV	Y	No $(E_{FL}\text{-}C_{55})_2$[c]	X-ray	4I3H	0.821
*Ab*Topo IV	Y	Y	X-ray	2XKK	0.979
*Kp*Topo IV	Y	Y	X-ray	6WAA	1.026
*Sp*Gyrase	No	Y	X-ray	4Z2C	0.836
*Sa*Gyrase	Y	Y	X-ray	5CDQ	0.734
*Ec*Gyrase	Y/N	Y	EM	6RKS/729C	1.198
*Mt*Gyrase	Y/Y	Y	X-ray	5BS8/7UGW	0.971/0.889
*Sc*Top2	N	N	X-ray	4GFH	1.124
hTOP2α	N	No C-gate	X-ray	4FM9	1.285
hTOP2α	Y	Y	EM	6ZY5	1.248
hTOP2β	Y	Y	X-ray	3QX3	1.158

[a] Root mean squares deviation is the average distance (in Å) between atoms of the superimposed structures.
[b] Core complex of *Sp* topo IV made up of dimers of the fused TOPRIM ParE30 and breakage-reunion ParC55 domains.
[c] A three-gate structure of Sp topo IV made up of dimers of the fused full-length ParE subunit and ParC55 domains.

Supplementary Table 4 Crystallisation conditions.

Method	Sitting-drop vapour diffusion
Plate type	MRC 96-well 2-Drop
Protein concentration	4.5 mg/mL
Buffer composition of the protein solution	20 mM Tris-HCl (pH 7.5), 200 mM NaCl and 0.05% NaN_3
Composition of the reservoir solution	50 mM sodium cacodylate, 62.5 mM KCl, 7.5 mM $MgCl_2$, 2.5% Tacsimate^{TM}, 5.5-7% isopropanol, pH 6.5
Volume and ratio of the drop	600 nl:400 nl protein: reservoir
Volume of reservoir	50 µl
Temperature for crystallization (K)	301

Supplementary Table 5 Diffraction limits and principal axes of ellipsoid fitted to diffraction cut-off surface from STARANISO[2] for the core complex with V18 and delafloxacin, grown in the presence of nucleant (8QMB).

Diffraction limits (Å) and corresponding principal axes of the ellipsoid fitted to the diffraction cut-off surface as direction cosines in the orthogonal basis (standard PDB convention), and in terms of reciprocal unit-cell vectors					
Principal axes	Diffraction limits	Reciprocal unit-cell vectors			Direction cosines in the orthogonal basis (standard PDB convention)
Diffraction limit #1	2.625	1.0000	0.0000	0.0000	0.894 _a_* - 0.447 _b_*
Diffraction limit #2	2.625	0.0000	1.0000	0.0000	_b_*
Diffraction limit #3	1.995	0.0000	0.0000	1.0000	_c_*

Supplementary Table 6 Diffraction limits and principal axes of ellipsoid fitted to diffraction cut-off surface from STARANISO[2] for the core complex with V18 and delafloxacin, grown in the absence of nucleant (8QMC)

Diffraction limits (Å) and corresponding principal axes of the ellipsoid fitted to the diffraction cut-off surface as direction cosines in the orthogonal basis (standard PDB convention), and in terms of reciprocal unit-cell vectors					
Principal axes	Diffraction limits	Reciprocal unit-cell vectors			Direction cosines in the orthogonal basis (standard PDB convention)
Diffraction limit #1	3.356	1.0000	0.0000	0.0000	0.894 _a_* - 0.447 _b_*
Diffraction limit #2	3.356	0.0000	1.0000	0.0000	_b_*
Diffraction limit #3	2.406	0.0000	0.0000	1.0000	_c_*

Supplementary Table 7 Diffraction limits and principal axes of ellipsoid fitted to diffraction cut-off surface from STARANISO[2] for the E18 structure 8C41.

Diffraction limits (Å) and corresponding principal axes of the ellipsoid fitted to the diffraction cut-off surface as direction cosines in the orthogonal basis (standard PDB convention), and in terms of reciprocal unit-cell vectors					
Principal axes	Diffraction limits	Reciprocal unit-cell vectors			Direction cosines in the orthogonal basis (standard PDB convention)
Diffraction limit #1	3.095	1.0000	0.0000	0.0000	0.894 _a_* - 0.447 _b_*
Diffraction limit #2	3.095	0.0000	1.0000	0.0000	_b_*
Diffraction limit #3	2.359	0.0000	0.0000	1.0000	_c_*

Supplementary references

1.Frisch, M.J. et al. Gaussian 16. Revision C.01. 2016. Gaussian Inc, Wallingford, CT, USA.

2.Vonrhein, C. et al. Advances in automated data analysis and processing within autoPROC, combined with improved characterisation, mitigation and visualisation of the anisotropy of diffraction limits using STARANISO.*Acta Crystallographica A-Foundation and Advances* **74**, A360-A360 (2018).

Najmudin et al_2024. NCOMMS-24-40126B_Fig. 3a_uncropped negative of agarose gel

Fig 3a. Delafloxacin-mediated pBR322 DNA cleavage is more efficient for *S. pneumoniae* topo IV than gyrase

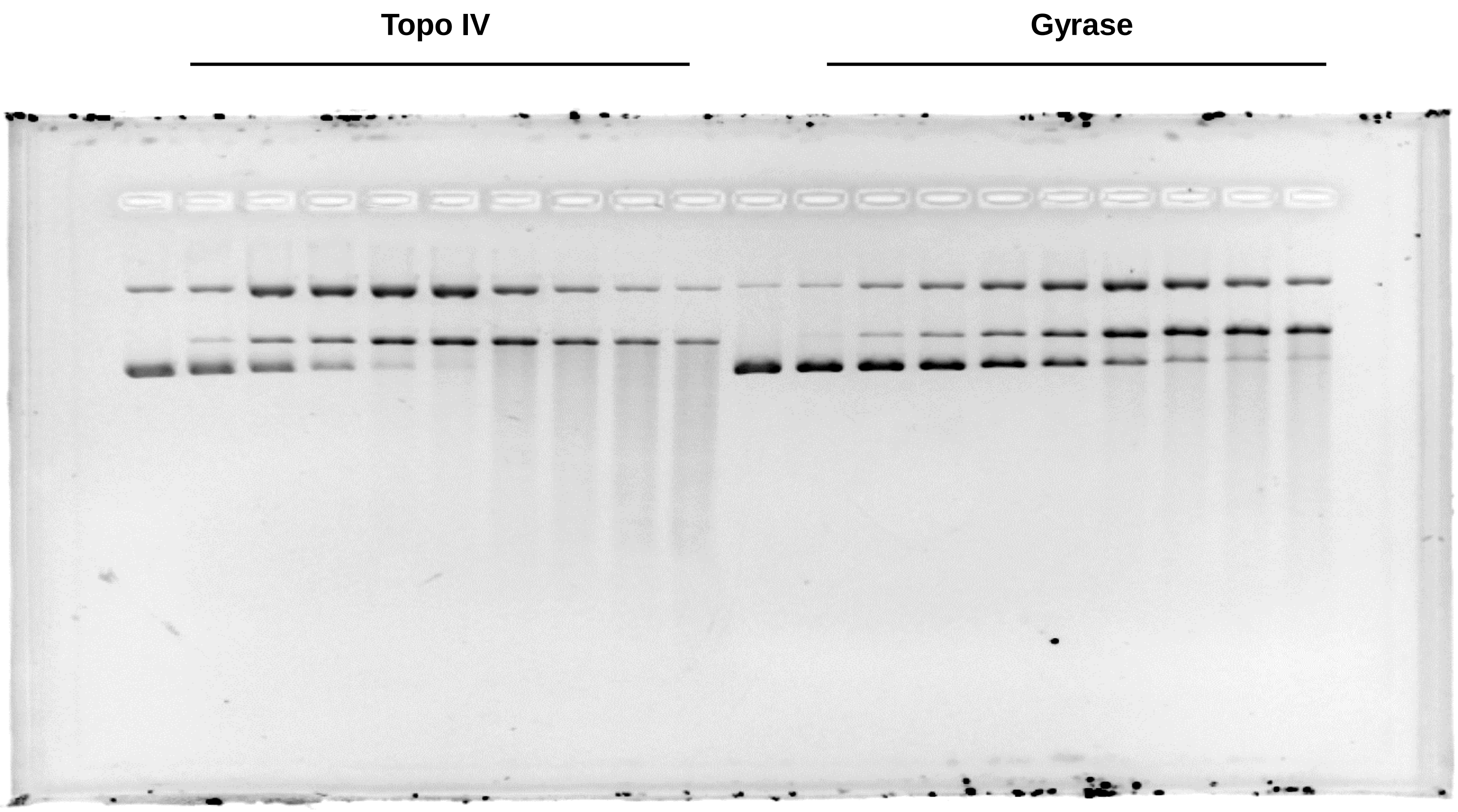

Najmudin et al_2024. NCOMMS-24-40126B_Fig. 3b_uncropped negative of agarose gel

Fig. 3b. ParE30-ParC55 fusion protein is active in pBR322 DNA cleavage and is targeted more efficiently by delafloxacin than by levofloxacin

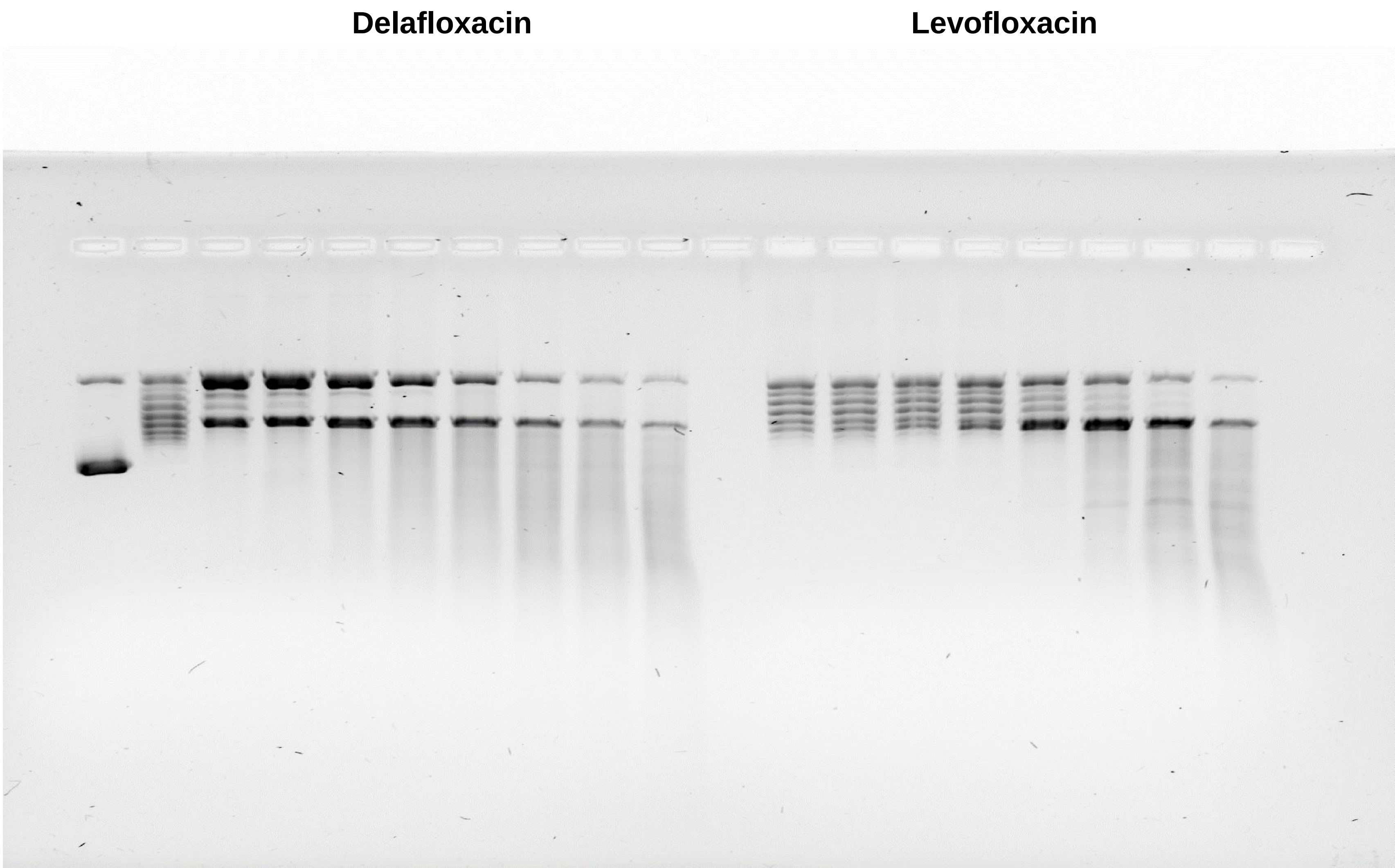

Supplement: Supplementary file 2 — Reporting Summary [file 41467_2025_60688_MOESM2_ESM.pdf]
