# Supplementary material for: Structural basis of topoisomerase targeting by delafloxacin
Source: Nat Commun. 2025 Jul 1;16:5829. doi: 10.1038/s41467-025-60688-3 (PMC12217528; doi:10.1038/s41467-025-60688-3)
Supplement: Supplementary file 3 — Description of Additional Supplementary Files [file 41467_2025_60688_MOESM3_ESM.docx]

**Description of Additional Supplementary Files for**

**Structural basis of topoisomerase targeting by delafloxacin**

**Supplementary Movie 1**

**Title:** Accompanying movie for supplementary figure 2 – DNA superposition

**Caption:** The V18 and E18 G-segment duplex DNAs adopt similar conformations in the topo IV complex stabilized by delafloxacin (in red). Magnesium ions are shown in purple. Both DNA strands are cleaved with the catalytic Y118 ParC residues (shown in orange) covalently linked one to each 5’-end of the 4-bp staggered breaks on complementary strands. DNA analysis with w3DNA software indicates that the V and E sites duplexes are bound in the cleavage complex as B-form DNA.

**Supplementary Movie 2**

**Title:** Accompanying movie for supplementary figure 6 – drug binding pocket

**Caption:** The N-1 heteroaromatic ring of delafloxacin is bound in a sub-pocket formed between DNA and ParE (8QMB). Protein residues and DNA are shown in surface representation, ParC in blue, ParE in yellow and DNA in grey. (See attached movie of the binding pocket).
